# Relationship between patient-reported disease severity in osteoarthritis and self-reported pain, function and work productivity

**DOI:** 10.1186/ar3121

**Published:** 2010-08-25

**Authors:** Alesia B Sadosky, Andrew G Bushmakin, Joseph C Cappelleri, David R Lionberger

**Affiliations:** 1 Pfizer Inc, Global Health Economics and Outcomes Research, 235 East 42nd Street, MS 235/9/2, New York, NY 10017, USA; 2 Pfizer Inc, Global Research and Development, 50 Pequot Avenue, MS 6025-B2275, New London, CT 06320, USA; 3 Southwest Orthopedic Group, 6560 Fannin St # 1016, Houston, TX 77030-2725, USA

## Abstract

**Introduction:**

Understanding the relationship between patient-reported osteoarthritis (OA) severity and other patient-reported outcomes in the real-world clinical setting can provide a basis for appropriate patient management. The objective of this study was to determine how patient-reported OA severity correlates with patient-reported outcomes including pain, function and productivity.

**Methods:**

We used the Adelphi Disease Specific Programme (DSP) for OA, a database aggregated from large, multinational, observational studies for specific chronic diseases. Data were obtained based on a 0 to 100 mm pain visual analogue scale (VAS) and a series of questions including functioning (that is, activities of daily living) and work productivity. OA severity was rated by the patients based on the question "How bad would you say your arthritis is now?" with potential responses of "mild," "moderate," and "severe." Regression models and chi-square analyses were used to evaluate the relationships between self-reported OA severity and other outcomes.

**Results:**

Of 998 subjects in the OA DSP U.S. database, 714 (72.5%) agreed to participate. This sample was predominantly female (61.7%) with a mean age of 63.8 ± 12.9 years. Increased OA severity was associated with an older population (*P *< 0.05). With increasing OA severity (mild, moderate, severe), statistically significant differences (*P *< 0.05) were observed in increased pain VAS scores (23.5, 50.2, 70.8, respectively), lower functioning outcomes, and a higher percent of overall work impairment due to OA (17%, 37%, 48%, respectively). The increased work impairment at greater severity levels also resulted in higher costs related to lost work productivity, with annual costs due to lost productivity estimated at $6,096, $13,2510, and $17,214 per patient for self-reported mild, moderate, and severe OA, respectively (*P *< 0.05 for pairwise comparisons).

**Conclusions:**

In the clinical practice setting, patient-reported OA severity was associated with other key patient-reported outcomes and thus may provide an accurate and tangible assessment of patients' perceptions of their disease. Identifying OA patients by their perceived severity level may be of benefit to patients and health-care providers when choosing treatment options aimed at reducing pain, and improving function and productivity.

## Introduction

Osteoarthritis (OA) is a degenerative joint disease that is characterized pathologically by loss of articular cartilage and concomitant development of osteophytes at the joint margins, and characterized clinically by pain, stiffness, fatigue, and functional impairment. These characteristics result in the substantial disability and reduced quality of life reported by patients with OA [1,2]. OA has been estimated to occur in 27 million individuals in the U.S. [3], and since age is the primary predictor, its prevalence is likely to increase as the proportion of older individuals in the population increases [4]. Although inflammation may occur, OA is not considered an inflammatory disease, and in the absence of both a clearly defined etiology and the availability of disease-modifying drugs, recommendations for OA management have consistently focused on reducing pain and improving function [5-9].

A variety of pharmacologic options are available for managing OA-related pain such as simple analgesics, nonsteroidal anti-inflammatory drugs (NSAIDs), oral corticosteroids, opioids, and injectables including corticosteroids and viscosupplementation with hyaluronan. Choosing among these medications is often determined by disease severity.

OA severity can be defined and graded using radiographic and other objective techniques for assessing OA pathology [10,11]. However, such severity may not correlate with patients' perceptions and therapeutic needs. For example, Johnson *et al. *[12] showed that self-reported improvement did not correlate with clinicopathologic findings including range of motion, disease activity, and radiographic grade.

In clinical trials, definitions of severity are generally based on cut-points for patient-reported pain, function, and global assessments. Two studies using 0 to 10 pain severity scales have suggested specific cut-points for pain in patients with OA [13,14]. One study identified the cut-points of 4 and 6 for patients with hip OA and 4 and 7 for those with knee OA [13], and the other study suggested that scores of 5 and 7 were the optimal cut-points [14]. However, in real world settings, categorizing patients as having mild, moderate, or severe disease based solely on pain cut-points may not necessarily provide a comprehensive and accurate assessment of OA severity from the patient's perspective. Factors such as functional impairment and worker productivity, while substantially affected by the presence of pain and pain exacerbations [15,16], may also contribute to a patient's overall perception of disease severity. A few orthopedic-specific rating scales have been designed to assess functional or physical limitations; however, they do not categorize severity levels to accurately guide physicians in their evaluation and treatment of patients.

The ability to characterize OA severity and its associated manifestations from the patient's perspective may provide a context within which management strategies may be determined and therapeutic outcomes evaluated. To our knowledge, there have been only limited attempts to characterize levels of OA severity using patient-based measures. These attempts have been either specific to orthopedic procedures [17-19], or lack the ability to measure patients' expectations on a full complement of disabilities [20-22].

A simple approach to establishing OA severity, with applicability to the real-world clinical setting, is for patients to self-rate their severity as mild, moderate, or severe. However, asking just one question on severity of OA needs to be supported by ascertaining whether such reporting of severity correlates with (or manifests via) other patient-reported outcomes that are interpretable, useful, and quantifiable. The objective of this study was to determine how several real-life parameters of patient perceptions of OA severity levels correlate with patient-reported pain, function, and productivity in clinical practice.

## Materials and methods

The Adelphi Disease Specific Programme (DSP) is a database that is aggregated from large, multinational, observational studies for specific chronic diseases [23]. The data were collected in clinical practice settings by physicians who provided relevant information on patients consulting for the disease of interest, with the patients being invited to participate in answering self-report questionnaires related to their symptoms, expectations, and health status. The current analysis is based on the DSP for OA for the year 2008 (OA DSP VII) which included data for subjects from the U.S. As this was a retrospective analysis of an existing dataset, local ethics committee approval was not required.

Severity of OA was rated by the patients based on the question "How bad would you say your arthritis is now?" with potential responses of "mild," "moderate," and "severe." Patients also reported on their demographic and disease characteristics, and outcomes data included a pain visual analogue scale (VAS; 0 to 100 with 0 = no pain and 100 = worst possible pain) to estimate OA-related pain severity during the past week, and questions on practical daily functioning that included items on ability to perform both basic and instrumental activities of daily living during the past week. These questions were adapted from Lawton and Brody [24] and scored on a 4-point Likert scale (1 = no difficulty, 2 = some difficulty, 3 = much difficulty, 4 = unable to do).

Additionally, data on productivity was captured using the Work Productivity and Activity Impairment scale (WPAI) [25]. The WPAI consists of six questions with the first question on employment status. The remaining five questions, referenced to the past seven days, pertain to hours missed because of OA; hours missed because of other reasons; hours actually worked; degree OA affected productivity while working (rating scale from 0 = no effect to 10 = completely prevented from working); and degree OA affected regular activities (rating scale 0 = no effect to 10 = completely prevented daily activities). By summing and dividing these responses accordingly, the percent work time missed due to OA (absenteeism) can be calculated, as well as the percent impairment while on the job due to OA (presenteeism), percent overall work impairments due to OA, and the percent activity impairment due to OA. These percentages were used to estimate the costs resulting from lost productivity at each level of OA severity based on average hourly wages in 2008 for all employees, seasonally adjusted, from the Bureau of Labor Statistics.

Descriptive analyses, regression models (adjusted for age and gender, with patient-reported severity of OA as the predictor), and chi-square contingency tables were used to evaluate the relationships between self-reported OA severity and other self-reported outcomes in order to quantify and construe their association with OA severity levels. In addition, as an ancillary analysis, a site-specific model was fit in which 10 binary variables, one for each joint, were added (to age, gender, and patient-reported severity of OA) along with the interaction of each joint with severity of OA. All analyses were pre-specified and performed using SAS version 9.2 (SAS Institute Inc., Cary, NC, USA). Evidence for statistical significance was based on a *P*-value less than 0.05.

## Results

A total of 998 subjects were in the OA DSP database from the U.S., and 71.5% (*n *= 714) agreed to participate. The demographic and disease characteristics of these 714 subjects (Table 1) show that the sample was predominantly female (61.7%), mean age was 63.8 ± 12.9 years, and 41% of patients were employed at least part-time. The mean time since first OA diagnosis was 5.6 ± 6.5 years, and the knee was the joint most frequently affected (72.4%). Severity of OA was rated as mild, moderate and severe by 36.7%, 47.2% and 16.1% of the 714 patients, respectively.

**Table 1:** Demographic characteristics of the population evaluated in the osteoarthritis Disease Specific Program (*N *= 714)

Variable (number of observations)	Value*
Mean age ± SD, years (*n *= 711)	63.8 ± 12.9
Age range, % (*n *= 711)	
18 to 44 years	7.9
45 to 64 years	40.9
≥65 years	51.2
Gender, % (*n *= 711)	
Female	61.7
Male	38.3
Employment, % (*n *= 674)	
At least part time	41.1
Unemployed	6.1
Retired	40.7
Student	0.3
Homemaker	9.4
Other	2.4
Mean time since diagnosis ± SD, years (*n *= 682)	5.6 ± 6.5
Joints affected, n (%)	
Neck	174
Shoulders	144
Elbows	56
Wrists	137
Metacarpophalangeal joints	141
Spine	280
Hips	214
Knees	505
Ankles	55
Feet	74

*Values and proportions are based on observed cases (that is, the number of subjects who supplied data for each variable).

**Figure 1:**
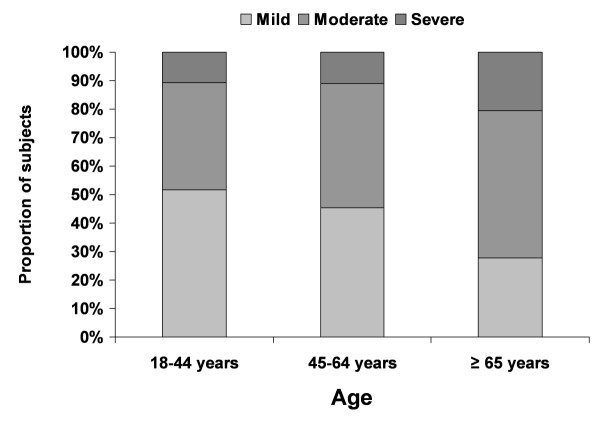
**Relationship between self-reported osteoarthritis severity and age**. *P *< 0.05 for this relationship based on chi-square analysis.

Time since first diagnosis increased with increasing OA severity: 4.6 years (95% confidence interval (CI): 3.8 to 5.3) for mild OA, 5.9 years (95% CI: 5.2 to 6.6) for moderate OA, and 7.2 years (95% CI: 6.0 to 8.4) for severe OA (*P *< 0.05 for each pairwise comparison adjusted for age and gender). Similarly, after adjusting for age and gender, increased age was associated with greater OA severity levels (*P *< 0.0001 from chi-square test; Figure 1). After adjustment for age and gender, mean scores on pain severity increased as OA severity increased: 23.5 (95% CI: 21.01 to 25.9) for mild OA, 50.2 (95% CI: 48.1 to 52.3) for moderate OA, and 70.8 (67.2, 74.4) for severe OA (*P *< 0.0001 for each pairwise comparison).

Greater OA severity resulted in increased levels of functional impairment, after adjustment for gender, as manifested by patient report of increased difficulty in performing a selected set of basic and instrumental activities of daily living (Table 2). For every item of functional ability, the difficulty score was greater than 1 at each OA severity level (lower bounds of the 95% confidence intervals were greater than 1), and the greatest difficulty was observed among patients who reported severe OA. Pairwise comparisons between OA severity levels additionally showed that differences in functional abilities were statistically significant (*P *< 0.05).

**Table 2:** Relationship between self-reported osteoarthritis severity and functional ability in performance of activities of daily living

Functional measure	Functional Score at Each Level of OA Severity (95% confidence interval)*		
	
	Mild	Moderate	Severe
**Basic activities of daily living**			
Dressing, including shoelaces and buttons	1.23 (1.16, 1.30)	1.44 (1.38, 1.50)	1.81 (1.70, 1.92)
Washing hair	1.13 (1.06, 1.20)	1.30 (1.25, 1.36)	1.53 (1.43, 1.63)
Rising from chair	1.34 (1.26, 1.41)	1.72 (1.65, 1.79)	2.11 (2.00, 2.23)
Getting in and out of bed	1.35 (1.27, 1.42)	1.70 (1.64, 1.76)	1.99 (1.88, 2.10)
Walking on flat ground	1.23 (1.16, 1.30)	1.59 (1.52, 1.65)	2.16 (2.05, 2.27)
Climbing five steps	1.43 (1.34, 1.51)	2.04 (1.96, 2.11)	2.49 (2.37, 2.62)
Washing and drying	1.09 (1.03, 1.15)	1.25 (1.20 (1.30)	1.52 (1.43, 1.60)
Taking a bath	1.23 (1.13, 1.32)	1.48 (1.40, 1.56)	1.92 (1.78, 2.06)
Toileting	1.17 (1.11, 1.24)	1.55 (1.49, 1.61)	1.88 (1.78, 1.98)
**Instrumental activities of daily living**			
Shopping	1.20 (1.13, 1.28)	1.56 (1.49, 1.62)	2.04 (1.92, 2.15)
Getting in and out of a car	1.25 (1.83, 1.33)	1.73 (1.67, 1.79)	2.15 (2.04, 2.26)
Performing chores (for example, vacuuming or gardening)	1.39 (1.30, 1.48)	2.00 (1.92, 2.08)	2.64 (2.50, 2.77)

Response options: 1-without any difficulty, 2-with some difficulty, 3-with much difficulty, 4-unable to do.

*All pairwise comparisons of arthritis severity categories were statistically significant (*P *< 0.05).

A site-specific model was used to evaluate the relationship between patient-reported OA severity and functional ability based on affected joints at three different body sites (Table 3). Estimates from this model were generally similar among the sites, and were also comparable with the overall model presented in Table 2, showing a trend with respect to an observed decrease in functional ability with increasing OA severity.

**Table 3:** Relationship between self-reported osteoarthritis (OA) severity and functional ability in performance of activities of daily living among patients with affected joints at three different body sites

Functional measure	Functional Score at Each Level of OA Severity								
	
	Mild			Moderate			Severe		
			
	Knees	Hips	Wrists	Knees	Hips	Wrists	Knees	Hips	Wrists
**Basic activities of daily living**									
Dressing, including shoelaces and buttons	1.28	1.31	1.43	1.60	1.68	1.70	2.22	2.08	1.97
Washing hair	1.20	1.17	1.26	1.49	1.63	1.55	2.01	1.89	1.94
Rising from chair	1.54	1.55	1.50	1.67	1.64	1.63	2.37	2.17	2.20
Getting in and out of bed	1.52	1.57	1.49	1.67	1.77	1.69	2.18	2.14	2.14
Walking on flat ground	1.39	1.48	1.32	1.51	1.52	11.48	2.29	2.05	2.12
Climbing five steps	1.62	1.60	1.45	1.91	1.96	1.78	2.65	2.49	2.57
Washing and drying	1.08	1.04	1.09	1.29	1.47	1.46	1.84	1.83	1.79
Taking a bath	1.42	1.33	1.41	1.68	1.79	1.73	2.08	1.98	2.05
Toileting	1.34	1.32	1.33	1.50	1.55	1.48	2.11	1.99	2.01
**Instrumental activities of daily living**									
Shopping	1.33	1.29	1.23	1.52	1.55	1.45	2.20	1.99	2.20
Getting in and out of a car	1.42	1.46	1.35	1.61	1.69	1.67	2.37	2.17	2.24
Performing chores (for example, vacuuming or gardening)	1.37	1.42	1.34	1.82	1.91	1.93	2.96	2.80	2.90

**Figure 2:**
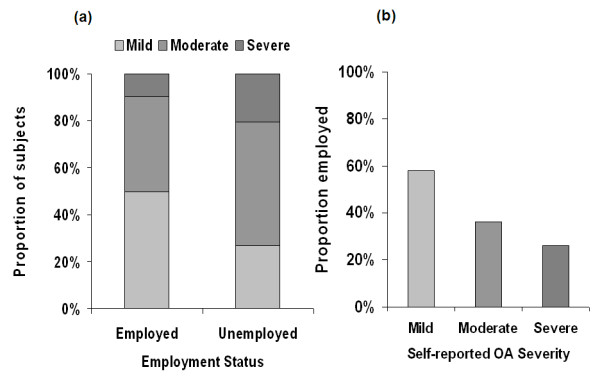
**Relationship between self-reported osteoarthritis (OA) severity and current employment status**. **(a) **Patient-reported OA severity stratified by employment status. *P *< 0.0001 based on chi-square analysis. **(b) **Proportion of patients at each OA severity level who reported being employed.

Patient-reported OA severity was significantly (*P *< 0.0001) associated with employment status (Figure 2). Higher proportions of unemployed patients reported moderate and severe OA relative to those who were employed (Figure 2a), and within each severity category, the proportion of employed patients decreased as severity increased (Figure 2b).

**Figure 3:**
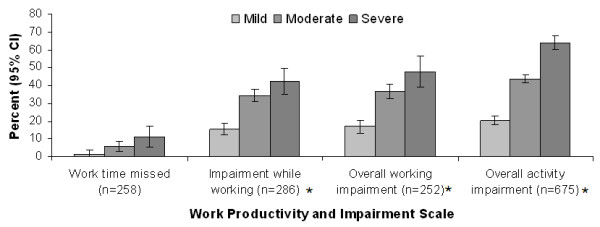
**Relationship between self-reported osteoarthritis (OA) severity and productivity**. Evaluation of productivity based on the Work Productivity and Activity Impairment (WPAI) questionnaire [25]. CI, confidence interval. Values of means of percent impairment were adjusted for age and gender. *P < 0.05 for pairwise comparisons between severity levels.

For all questions evaluating the percent of OA-related impairment of work and activity, increased impairment was reported at greater OA severity levels (*P *< 0.05) (Figure 3). All pairwise comparisons, adjusted for age and gender, showed significant differences (*P *< 0.05) in work impairment between OA severity levels except for percent work time missed due to arthritis between moderate and severe OA (*P *= 0.09).

**Figure 4:**
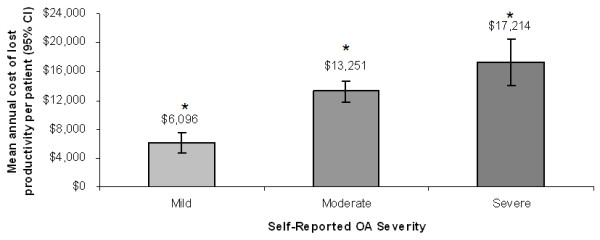
**Relationship between self-reported osteoarthritis severity and mean overall cost of lost productivity per patient per year**. *P *< 0.05 for all pairwise comparisons.

Work impairment among employed individuals also resulted in overall costs of $9,958 per patient per year resulting from lost productivity. When stratified by self-reported OA severity after adjusting for age and gender, lost productivity costs were significantly higher with increasing levels of severity. These costs were estimated at $6,096 per year for a patient with mild severity, $13,251 for moderate OA severity, and $17,214 for a patient with severe OA (Figure 4, *P *< 0.05 for all pairwise comparisons).

## Discussion

We can consider that defining disease severity from the patient's perspective may be a relevant strategy for the daily clinical management of patients with OA. For such a strategy to be effective, it is important to establish that levels of patient-reported disease severity do in fact demonstrate a relationship with disease-related outcomes. This study indicated that patients with OA who reported their disease severity as being mild, moderate, or severe, also reported correspondingly greater levels of pain, functional impairment, and productivity impairment. For each increasing level of OA severity, the corresponding magnitude of the outcome was significantly and typically different from that reported for the other OA severity levels, with pain, function, and work productivity most impacted in patients who rated their OA as severe.

Pain and function are core symptomatic outcomes of OA that are frequently targeted as part of pharmacologic therapy, and it may be expected that these outcomes are associated with patient perceptions of OA severity. However, it was also of particular interest to note that the results for productivity were in accord with the other outcomes, since a previous study suggested an association among pain, function, and economic productivity in patients with OA [16]. In the current study, not only did productivity show a significant association with severity, with pairwise comparisons between severity categories demonstrating statistical significance for virtually all WPAI items, but the interference with productivity was substantial in patients with severe OA. At this level of severity, approximately three-quarters of the patients (74.1%) reported being unemployed, and for those who were employed there was 42% impairment while working, and overall working impairment was 47%. These data are consistent with published reports that productivity losses substantially contribute to the economic burden of OA [26-29], despite the fact that OA is more prevalent in an older population who may not necessarily be expected to be employed.

This study also suggests that there are substantial indirect costs related to lost productivity in a patient with OA (mean costs of $9,958 per year). Importantly, these costs were significantly higher at greater OA severity with the annual cost for a patient with severe OA almost three times that of a patient with mild OA. These results are not only important from the economic perspective, but offer further evidence that the patient's perception of OA severity may facilitate assessment of functional and economic outcomes.

In contrast to other available measures [20,22,30], patients were asked to characterize their OA severity and we then evaluated the association between their response and external measures. Although such an approach does not enable a quantitative measure of severity, it provides a patient-based perspective that demonstrates significant associations with other patient-reported outcomes.

It should also be noted that this study focused on the associations between a simple patient-reported assessment of OA severity and other patient-reported outcomes. While the goal was to enhance our understanding of OA by relating patients' perceptions of severity to other measures of interest, it also provides insight into what it means from the patient's perspective to have mild, moderate, or severe OA.

A similar patient-based perspective in patients with OA was evaluated in a study by Reichmann *et al. *[31] in which patients were asked to rate their overall health status as excellent, very good, fair, or poor. Our assessment was based on a question that asked patients to specifically rate their OA severity as mild, moderate, or severe. Patients with OA are generally characterized by a substantial presence of comorbid conditions, some of which are associated with additional disability and functional limitations [32,33]. Thus, the generic question on overall health status is expected to encompass a multitude of factors including but not restricted to the patient's OA.

While in the present study it cannot be totally excluded that patients may have considered other factors to some extent when rating their OA severity, the presence of comorbidities would be expected to contribute substantially, to a large extent, to patients' perceptions of their health status. In fact, Reichmann *et al. *found that, among patients with knee OA, self-reported health status was associated with comorbidity and, in addition, functional status. Although a moderate association between patient-rated OA severity and patient-rated general health status is also to be expected, we believe that such a correlation will not be large as patient-reported OA severity (though related to) is distinct from patient-reported general health status, and represents a measure that may be useful in the clinical setting for enabling disease-specific treatment decisions.

Importantly, this investigation is not a validation study and does not attempt to provide detailed psychometric evaluation of the assessment under consideration, a subject that is beyond the scope of this research and the data presented. Similarly, no cause-and-effect relationships can be drawn from this cross-sectional observational study, and findings are limited to the strength and magnitude of the observed associations.

Several limitations of this study should be considered, including the fact that it was based on physicians' and patients' agreement to participate. It is possible that individuals who participated may have characteristics and perceptions different from those who refused to participate, thereby introducing selection bias and reducing the generalizability. The introduction of recall bias is also a common limitation of many studies based on questionnaires. However, this bias was minimized by using questions with a maximum recall period of the past seven days. While the cross-sectional nature of DSPs precludes any causation, no cause and effect imputation was made for the ratings of severity of either OA severity or other outcomes. Any relationships should be considered associative rather than causal.

That neither the type of employment nor the specific site of diagnosed OA were captured in the questionnaire may also be considered a limitation, since the former is likely to affect the absolute magnitude of productivity loss, and the latter is likely to variously affect functionality with regard to activities of daily living. In lieu of a site-specific OA diagnosis, an analysis of function was performed based on affected joints, and was observed to be consistent with the overall model. However, it should be noted that the site-specific results should be interpreted cautiously; the number of variables complicates the model and its interpretation, and the presence of multiple affected joints in a proportion of patients is also likely a confounding factor. Further evaluation of OA severity based on these variables would provide interesting supplementary information on the relationship between OA severity and outcomes.

Another limitation is that we did not control for the potential effects of comorbid conditions on the patient's perception of OA disease severity. However, the consistency of results across outcomes, including the narrow range of variance, suggests that this impact was low. With regard to the diagnosis of OA for inclusion, this diagnosis is dependent on the diagnostic skill of the treating physician, and it is therefore possible that misdiagnosis may have occurred in a small proportion of the sample population. This study could also be criticized for not comparing patient-reported severity with radiographic results. However, radiographic observations are physician-reported outcomes and their practicality for making clinical treatment decisions such as for knee replacement is unclear and may be better determined by functional status and patient preferences [34]. Nevertheless, demonstrating whether an association exists between radiographic and patient-reported OA severity can help confirm the value of using patient-reported assessment.

Despite these limitations, we suggest that the approach described here enables a rapid assessment of OA severity that may be of value in the clinical setting for providing an accurate, appropriate, and quantifiable measurement of the patient's perceived health status, especially with respect to symptoms. This metric provides a practical comparison, utilizable among practice specialties (family practice, rheumatology, orthopedics, and so on), for providing a better understanding of how patient's may perceive changes in their OA severity. Additionally, for second and third party payers, it may potentially provide a measure of efficacy on patients' risk pool for future disease expectations. A more rigorous evaluation of this technique will also help integrate the patient's perspective into an overall definition of OA severity.

## Conclusions

The significant associations between self-reported OA severity and other patient-reported outcomes indicate the clinical relevancy of asking patients to self-evaluate their OA severity. This simple and direct approach for determining OA severity represents a practical solution in the clinical setting that may benefit health care providers when choosing treatment options aimed at reducing pain and improving patient function and work productivity. Further analyses of these relationships and evaluation of patient-reported severity with other clinically relevant criteria may help confirm the utility of this method of defining and assessing OA severity in clinical practice.

## Abbreviations

DSP: disease specific programme; NSAIDs: non-steroidal anti-inflammatory drugs; OA: osteoarthritis; OMERACT/OARSI: outcome measures in rheumatology/Osteoarthritis Research Society International; VAS: visual analog scale; WOMAC: Western Ontario and McMaster Universities index of osteoarthritis; WPAI: work productivity and activity impairment scale.

## Competing interests

Joseph C Cappelleri, Andrew G Bushmakin, and Alesia Sadosky are employees of Pfizer Inc, the sponsor of this study. David R Lionberger has served as a consultant for Pfizer Inc., Aesculap, Smith & Nephew, King Pharmaceuticals, and Zimmer. Dr. Lionberger was not financially compensated for his participation in this project.

## Authors' contributions

All authors contributed to the study design, analysis, interpretation of results, and critical review of the manuscript.
